# How Not to Do WLS Fitting in Calibration with Heteroscedastic Data

**DOI:** 10.1021/acs.analchem.5c07874

**Published:** 2026-04-13

**Authors:** Joel Tellinghuisen

**Affiliations:** Department of Chemistry, Vanderbilt University, Nashville, Tennessee 37235, United States

## Abstract

In the least-squares fitting of data of varying precision to functions that are algebraically linear in the adjustable parameters, the correct weights for obtaining optimal results are the pointwise inverse variances σ_*i*_^–2^. These have often been obtained from the sampling statistics of replicate measurements of the uncertain quantity *y*, a procedure that has come to define weighted least-squares (WLS) calibration fitting. Unfortunately, such variance estimates are notoriously imprecise, and in a long-ago Monte Carlo study involving a linear response function and weights ranging over a factor of 5, ordinary least squares (OLS) outperformed WLS for almost every tested set of calibration *x* values and replicates. But there is a better way of obtaining the weights: variance-function estimation. This method relies on the fact that the variances for most physical data follow simple, smooth functions having two or three terms proportional to a constant, *y*, and *y* ^2^. Since the replicate data are used collectively to estimate the variance function, the number of statistical degrees of freedom is large enough to achieve good precision in the calibration weights. When variance-function weighting is included in the aforementioned OLS-vs-WLS comparisons, it wins over OLS for almost every combination with *m* = 4–8 *x* values and *n* = 3–5 replicates. Further, of the four ways of weighting in the variance-function fitting, the iterative reweighting method gives the best results, in support of earlier findings.

## Graphical abstract

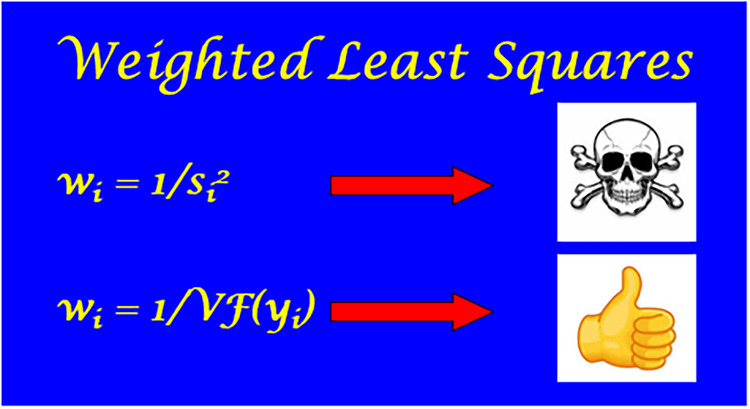

## Introduction

In analytical work, calibration curves are used to relate the concentration or amount of an unknown to appropriately selected samples considered “known.” Such curves are often linear, *y* = *a* + *bx*, and the calibration parameters, *a* and *b*, are commonly obtained using the method of least squares to fit the signals *y* obtained for the samples of known *x* (calibrants). If the signals vary in precision (are heteroscedastic), weighted least squares is required for optimal estimation. To obtain the needed weights, many authors in recent works have resorted to methods based on “goodness of fit” tests. However, such methods are seriously flawed.^1^ The proper weights are the inverse variances of the *y* values.^2^ To obtain these variances, workers have mostly used the statistics of replicate samples, often as few as 3 at each *x*; and WLS has generally come to refer to this procedure in works on calibration. However, already in 1973, Jacquez and Norusis showed that this approach can be worse than ordinary least squares (OLS),^3^ which assumes the data are homoscedastic, hence all weighted the same (*w* = 1). The problem is that sampling-based variance estimates have high relative uncertainty (2/ν)^1/2^, where ν is the degrees of freedom—which is 1 less than the number of points *n* for sampling statistics. This is 100% for *n* = 3, so obviously, WLS must perform worse than OLS for data that are truly homoscedastic. In the work of ref 3, OLS continued to outperform WLS for most combinations of *m* = 4–10 calibration points and *n* = 2–10 replicates for a case where the heteroscedasticity gave weights spanning a range of 1–5.

There is, however, a better way of estimating the weights from sampling data: variance-function estimation.^4–6^ Physical data commonly exhibit variances that vary smoothly with *y*, following functions that contain 2 or 3 terms, proportional to a constant, *y*, and *y* ^2^. This dependence was shown for spectrophotometry half a century ago.^7^ Thus, for example, 3 reps at each of 6 *x* values gives ν = 16 for a 2-parameter VF; and by definition, the weights vary smoothly with *y*. In ref 1, Monte Carlo simulations on a model like this showed only 3% precision loss in estimating the calibration parameters *a* and *b*.

## Methods

Jacquez and Norusis (JN) used Monte Carlo simulations on 400–1500 data sets to obtain their results.^3^ Here, I use the same approach to include variance-function-based weights in the comparisons, on 40,000 simulated data sets for each computation. This gives a precision of 0.35% [relative standard deviation = (2ν)^−1/2^] for the Monte Carlo-estimated parameters, which is a factor of 10 better than for JN’s *n* = 400. I consider only a model of theirs that gave near-total wins for OLS over WLS (their Table 1). The true values of *a* and *b* are both 1, with σ_*i*_ = *x*_*i*_/2 + 3 and *x*_*i*_ values ranging 1–10: {*x*_*i*_} = {1, 4, 7, 10}, {1, 2, 4, 7, 9, 10}, {1, 2, 4, 5, 6, 7, 9, 10}, and {1–10}. JN considered *n* = 2, 3, 4, 5, and 10 replicates at each *x*_*i*_; I include just *n* = 3, 4, and 5 reps and *m* = 4–8 calibration points, by which point the better performance of variance-function weighting is clear. I give precision comparisons for the estimates of the calibration parameters *a* and *b*.

The Monte Carlo simulations were done as in previous works:^1,5,6^ Random, Gaussian error of magnitude σ_*i*_ = 3 + *x*_*i*_/2 was added to the true *y*_*i*_. Variance estimates *s*_*i*_^2^ from the replicates were obtained from simple statistics, *s*_*i*_^2^ = Σ (*y*_*i*_–*y̅*)^2^/(*n*–1), where *y̅* is the average of the *n* replicates at *x*_*i*_. The *s*_*i*_^2^ values were then fitted to the variance function, VF­(*x*) = (*c* + *dx*)^2^, giving estimates of *c* and *d* for each simulated data set. Finally, the weights *w*_*i*_ = 1/VF­(*x*_*i*_) were used to fit the data to the calibration response function, *y* = *a* + *bx*. The conventional “WLS” results were obtained weighting with the sampling estimates, *w*_*i*_(WLS) = 1/*s*_*i*_^2^. The calibration fitting included all replicates rather than averages at each *x*_*i*_. For each weighting, the precision loss was obtained by comparing the Monte Carlo standard deviation SD with the true standard error SE, as obtained from the covariance matrix for weighting with the true VF. For linear least-squares models, these SEs are exact.

There is an additional complication in the variance-function fitting. The variance estimates have relative uncertainty (2/ν)^1/2^, as already noted [i.e., σ­(σ_*y*_^2^) = (2/ν)^1/2^σ_*y*_^2^]. This means that in the least-squares estimation of the VF, the *s*_*i*_^2^ estimates should be weighted as

1 $$w\left(\mathrm{VF}\right)=\left(v/2\right)\sigma_{y}^{-4}$$

But what to use for σ_*y*_ here? There are at least four choices:^1^ (1) Use the sampling *s*_*i*_. (2) Fit ln­(*s*_*i*_^2^) to ln­(VF). Since σ­(ln*y*) = σ_*y*_/*y*, the weights now become ν_*i*_/2 and are all the same if all *s*_*i*_^2^ values come from the same number of replicates. (3) Weight as 1/VF^2^. This requires iterative reweighting since the VF changes with each iteration until convergence is achieved (usually within 10 cycles). (4) Incorporate the VF^–2^ weights directly in the minimization target

2 $$S=\Sigma w_{i}\delta_{i}^{2}=\Sigma \frac{{\left({\mathrm{VF}}_{i}-s_{i}^{2}\right)}^{2}v_{i}}{2{\mathrm{VF}}_{i}^{2}}$$

where δ_*i*_ is the difference between the calculated and observed values. In method 4, the VF parameters are estimated as part of the normal nonlinear least-squares iterations. Method 3 is similar to the effective variance (EV) method used for models with uncertainty in multiple variables, and method 4 is analogous to an EV variant I have labeled EV_2_.^8^ In the tests in ref 1, method 3 performed best and method 1 worst.

An alternative to VF fitting is SD fitting. Although the SD is a biased estimator,^5^ the Monte Carlo simulations show that the VF parameters are anyway biased, with only weighting method 3 giving acceptably small bias. Like variance estimates, SD estimates have relative uncertainty, given above as (2ν)^−1/2^, or a factor of 2 smaller than that for variance. Thus, e.g., for the SD version of eq 2, VF_*i*_ is replaced by SD_*i*_, *s*_*i*_^2^ becomes *s*_*i*_, and the factor of 2 is moved to the numerator. With the SD version of eq 2, there is less Monte Carlo variation in the terms in the sum, which in turn leads to fewer divergences in the simulations. Of course, the proper weights in the calibration fitting now become *w*_*i*_ = 1/[SD­(*x*_*i*_)]^2^.

The computations were performed using programs in Microsoft Fortran and KaleidaGraph.

## Results and Discussion

In this discussion, I will use WLS for conventional 1/*s*_*i*_^2^ weighting as distinguished from VF weighting. I first use the test model to compare the four methods of weighting the 1/*s*_*i*_^2^ values in the VF estimation fitting. These will be designated as VF1 to VF4. The VF2 method (log fitting) gave no divergences, but the other VF methods gave 10–30 nonconvergences in 40,000 simulations, up to 79 (0.2%) for one VF3 simulation. These cases were omitted from the Monte Carlo statistics; they mostly involved opposite signs for *c* and *d* that led to anomalously small VF and single huge terms in the minimization target sums. SD fitting gave only a few divergences. In the fitting of the calibration data to the linear response function, there were only incidental convergence failures for all three methods.

Figure 1 shows the percent imprecision increase in the estimation of the calibration slope *b*. For *n* > 3, *s* ^–4^ weighting is clearly the worst, and VF3 weighting is generally the best; but the differences for methods 2–4 are not very significant. Note that the sometimes greater precision *loss* for more *x*_*i*_ values does not mean that the actual precision is lower. Thus, for example, the VF3 precision loss for 3 reps is the greatest for 8 *x*_*i*_ values, but the true slope SEs for *m* = 4, 6, and 8 are 0.476, 0.394, and 0.377, respectively, with Monte Carlo estimates of 0.523, 0.427, and 0.416.

**Figure 1:**
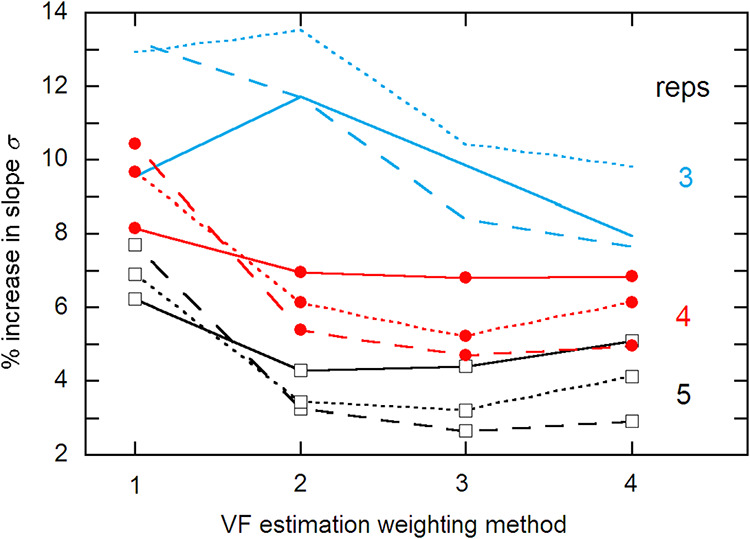
VF estimation weighting dependence of the precision loss in the Monte Carlo estimation of the calibration slope *b*, for *n* = 3–5 reps and *m* = 4–8 *x*_*i*_ values, identified by line style: 4 – solid, 6 – dash, 8 – dotted. The reference is the true σ_*b*_ for each *m*,*n* combination.

Figure 2 shows the SD function slope parameter *d* as a function of the weighting method. As was found in ref 1, VF3 weighting gives results closest to true, with all 9 estimates nearly identically equal to the true value. Similar behavior is found for the SD intercept *c*, but with more scatter and with the VF3 estimates averaging about 7% below the true value of 3. In a rough sense, the different weighting methods scale the SD function up or down, affecting the calibration parameters only moderately, since perfect weight scaling yields identical parameters.

**Figure 2:**
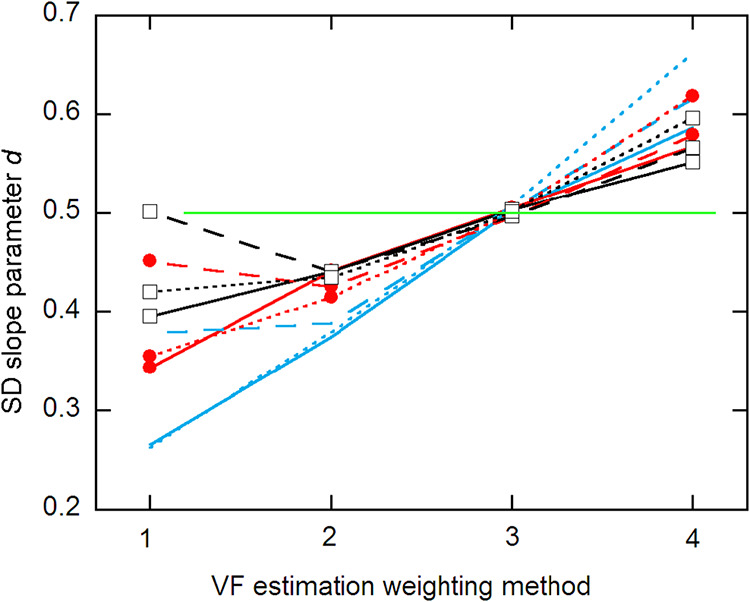
Estimates of SD function slope *d* for different VF estimation weighting methods. The results follow the same labeling as in Figure 1. The horizontal line marks the true value, *d* = 0.5.

Figure 3 compares the imprecision in the estimation of the calibration slope for WLS, OLS, and VF3 fitting. For these *m,n* combinations, WLS is marginally better than OLS for just the 4,5 example. Similarly OLS is better than VF3 for just the 6,3 case. Results for the calibration intercept *a* are similar: WLS outperforms OLS in two cases, and VF3 betters OLS in all cases.

**Figure 3:**
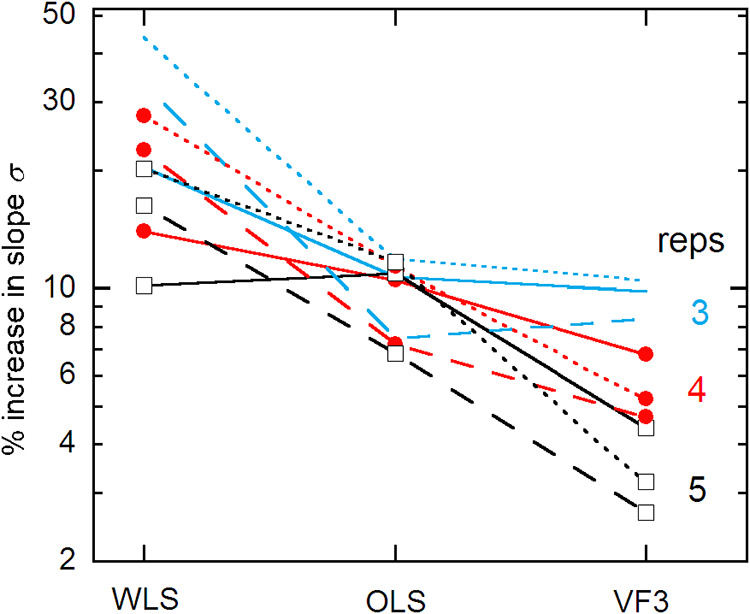
Precision loss in the estimated calibration slope *b* for three methods of fitting the data. Results are displayed for the same *m,n* combinations as in Figures 1 and 2.

There is one more important issue to address: What does the analyst report for the uncertainties in the calibration parameters (and the unknown)? In almost all least-squares programs, the SEs (called parametric) are taken from the covariance matrix. With incorrect weighting, these will be biased. For the present comparisons, the parametric estimates were taken as the root-mean-square (rms) values from the Monte Carlo estimates of the covariance matrix. For several *m,n* combinations, the Monte Carlo and parametric estimates of σ_*b*_ agreed within 10% for both VF3 and OLS, but the parametric values were ∼30% low for WLS. For σ_*a*_, the parametric estimates agreed within ∼3% for VF3, but were ∼30% high for OLS and ∼25% low for WLS. In short, only VF3 gives adequate parametric SE estimates in these comparisons.

## Conclusions

Weighted least-squares fitting should be done with VF-estimated weights to ensure optimal results in the fitting of heteroscedastic data in cases where the data variance function can be expected to depend smoothly on experimental parameters, which is true for most physical data. Although Jacquez and Norusis took their SDs to be functions of *x*, they should normally be dependent on *y*, since that is the quantity assumed to have measurement uncertainty. In the VF fitting, it is then appropriate to take the replicate average *y* as the independent variable. However, in calibration fitting with linear response functions, *y* is often nearly proportional to *x*, resulting in negligible difference between these choices of independent variables in the VF.

An important argument for expressing the VF as a function of the measured quantity is that in actual application, it is not necessary to re-estimate VF­(*y*) for each new data set because it is a function of the instrumentation and experimental protocol. Accordingly accumulated replicate data from day-to-day work can make VF­(*y*) nearly exact. This is shown in Figure 2 of ref 6 for the abundant gas chromatographic data of Zorn et al.^9^ (who did use VF weighting, but chose to obtain the VFs separately for each data set as functions of *x*). Another example is the determination of a spectrophotometric VF as a function of wavelength and absorbance.^10^ In fact, this is an example of multivariate fitting, and VF­(*y*) will apply equally well to multivariate calibration, as long as *y* remains the only uncertain variable. In one more example, the computational details of VF estimation are illustrated for both KaleidaGraph and Excel in Supporting Information, for the fit of LC-MS-MS data for cocaine and naltrexone from Desharnais et al.^11^ to a common VF.

A question often asked in WLS is how many replicates are enough? The results in JN’s Table 1 show that even 10 reps provide at most ∼5% better calibration slope precision than OLS, in conventional WLS, with *w*_*i*_ = 1/*s*_*i*_^2^. (That result is for *m* = 4 *x*_*i*_ values). On the other hand, Figure 3 shows that VF weighting gives only ∼4% slope precision loss for 5 reps, which is a factor of 2 smaller than for OLS. For the more strongly heteroscedastic example in ref 1, the loss was just 3% for *m,n* = 6,3; and an incorrect but similar VF gave negligible difference.

Although VF weighting beats OLS in almost all cases in the present comparisons, the actual (Monte Carlo) OLS precision losses and the errors in their parametric estimates are small enough to be unimportant in many applications. The reason is the small range (1–5) of the weights; so weighting can be expected to be relatively unimportant in other cases where the weight spread is this small or smaller. JN’s other examples involved weight spreads of 1–10 (their Table 2 and 1–100) (Table 3); and in those cases, conventional WLS (1/*s*_*i*_^2^) weighting outperformed OLS in 35% and 75% of the cases, respectively, for calibration slope estimation. As a general guideline, weight spreads greater than 1–10 do demand weighting, and VF weighting is needed to achieve the best precision and reliable parametric estimates of the SEs.

All of JN’s and the present results have assumed normally distributed random error in the data, which can be hard to prove for actual data. However, concern with this problem is generally overblown because (1) data are often collected with instruments that average multiple readings and (2) with replicates, calibration data sets often include 20 or more points. In both cases, the central limit theorem ensures near normality of the data in (1) and of the fit parameters in (2) even if the data are not normal, as long as they have finite variance.

Do these results apply as well to nonlinear least squares? It cannot be proved that inverse-variance weighting gives minimum-variance estimates of parameters in nonlinear models, in part because many nonlinear estimators do not even have finite variance. However, it is generally accepted that such weighting does give optimal or near-optimal results for confidence limits (which can be obtained even for parameters with infinite variance); and this has been demonstrated for specific models.^12^ Thus, VF estimation remains important for nonlinear fitting. Also, inverse-variance weighting remains rigorously optimal for many nonlinear response functions, like polynomials of order >1, because such functions are linear in the adjustable parameters, so remain in the linear least squares tent.

## Supplementary Material



## Notes

Portion of KaleidaGraph data sheet for VF2 and VF3 analysis of LC-MS-MS data for cocaine and naltrexone; results for VF2 analysis with 3 adjustable parameters (red) and 2 (blue); VF3 analysis (iterative reweighting); VF4 analysis; abbreviated excel worksheet for VF3 analysis of data (PDF)

## References

[1] Tellinghuisen J. (2022). Goodness-of-Fit
Tests in Calibration: Are They Any Good for Selecting Least-Squares
Weighting Formulas?. Anal. Chem..

[2] Aitken A. C. (1936). On least
squares and the linear combination of observations. Proc. R. Soc. Edinburgh.

[3] Jacquez J. A., Norusis M. (1973). Sampling experiments on the estimation
of parameters
in heteroscedastic linear regression. Biometrics.

[4] Davidian M., Carroll R. J. (1987). Variance Function
Estimation. J. Am. Stat. Assoc..

[5] Tellinghuisen J. (2008). Least squares
with non-normal data: Estimating experimental variance functions. Analyst.

[6] Tellinghuisen J. (2019). Calibration:
Detection, Quantification, and Confidence Limits Are (Almost) Exact
When the Data Variance Function is Known. Anal.
Chem..

[7] Ingle J. D., Crouch S. R. (1972). Evaluation of Precision of Quantitative Molecular Absorption
Spectrometric Measurements. Anal. Chem..

[8] Tellinghuisen J. (2018). Least-Squares
Analysis of Data with Uncertainty in *y* and *x*: Algorithms in Excel and KaleidaGraph. J. Chem. Educ..

[9] Zorn M. E., Gibbons R. D., Sonzogni W. C. (1997). Weighted least squares
approach to
calculating limits of detection and quantification by modeling variability
as a function of concentration. Anal. Chem..

[10] Tellinghuisen J. (2000). Statistical
Error Calibration in UV-Visible Spectrophotometry. Appl. Spectrosc..

[11] Desharnais B., Camirand-Lemyre F., Mireault P., Skinner C. D. (2017). Procedure
for the
Selection and Validation of a Calibration Model I — Description
and Application. J. Anal. Toxicol..

[12] Tellinghuisen J. (2003). A study of
statistical error in isothermal titration calorimetry. Anal. Biochem..

