# Supplementary material for: How Not to Do WLS Fitting in Calibration with Heteroscedastic Data
Source: Anal Chem. 2026 Apr 13;98(16):11641–4. doi: 10.1021/acs.analchem.5c07874 (PMC13130157; doi:10.1021/acs.analchem.5c07874)
Supplement: Supplementary file 1 [file ac5c07874_si_001.pdf]

Supporting Information for

How Not to Do WLS Fitting in Calibration with Heteroscedastic Data

Joel Tellinghuisen,*

Department of Chemistry, Vanderbilt University, Nashville, Tennessee 37235, United States

Email: joel.tellinghuisen@vanderbilt.edu. Phone: (615) 322-4873. Fax: (615) 343-1234

ABSTRACT

Variance function estimation is illustrated for LC-MS-MS data from Desharnais, *et al.*,[11] using the KaleidaGraph and Excel programs. The data for two analytes, cocaine and naltrexone, fit a common variance function (VF) when the variances are expressed as a function of the measured signals. This example served also as the basis for Figures 6 and 7 in ref 1 (but without computational details). VF estimation methods 2-4 are shown for KaleidaGraph, methods 3 and 4 for Excel. (See also ref. 8 for more details on such computations.)

TABLE OF CONTENTS

KaleidaGraph Methods p S-1

Excel Methods p S-4

References p S-5

KaleidaGraph Methods. KaleidaGraph (KG) is a column-oriented program, as opposed to cell-oriented Excel. The **General** least-squares (LS) fitting algorithm is used for both linear and nonlinear models and requires the user to specify the functional relation between the x and y variables. The fitting is graphically oriented so only two dimensional fits are covered directly. However, additional dimensions can be handled by displaying fit residuals as a function of the index number of the point in the dataset. This is needed for the VF4 method. The program uses the Marquardt method and provides standard error estimates (**Errors**) from the covariance matrix – the only KG fitting routine to do so.

The form assumed in ref 1 for the VF for the data from ref 11 is a sum of squares expressing constant and proportional error contributions to σ_y^2,

$$\mathrm{VF}(y) = c^2 + (dy)^2 , \tag{S-1}$$

as opposed to the square of a standard deviation function used by Jacquez and Norusis.[3] The variance estimates s_i^2 all come from 5 replicates so have relative uncertainty $(2/4)^{1/2}$. Thus the uncertainties for $\ln(s_i^2)$ are all $2^{-1/2}$, so unweighted fitting can be used in method 2. When weights are to be used in KG, they are provided as a column of σ values. This can be seen in a portion of the data sheet in Figure S-1, where column **C4** contains the σ(ln(var)) values. The **ln(var)** values are plotted vs. **y mean**, the **General** fit is selected under the **Curve Fit**

menu, and a fit name (**New Fit** by default) is entered by selecting **Edit General...** . Selecting this name will open a dialogue containing a **Define...** button, in which the **y** column is selected, the **Weight Fit** box is checked, and the fit relation (quantity to the right of y =) is entered, namely

$$\text{ln(c^2 + b*x^f); c=.002; b=.02; f=2 ,} \qquad \text{(S-2)}$$

which includes initial values for the adjustable parameters. This particular fit contains 3 parameters, including a variable power for x (f). Results show that f is well within its uncertainty of the expected power 2, so the fit is rerun with f replaced by 2 and b by d^2. Results are in Figure S-2.

	mean y C0	y var C1	sig var C2	ln(var) C3	s-ln C4	indx C5	F=0 C6
0	0.12900	5.50e-06	8.1833e-06	-12.111	0.70711	0	0.0000
1	0.24940	1.83e-05	2.3594e-05	-10.909	0.70711	1	0.0000
2	0.32860	0.000116	3.9078e-05	-9.0593	0.70711	2	0.0000
3	1.2350	0.000177	0.00051846	-8.6422	0.70711	3	0.0000
15	3.5270	0.0111	0.0042103	-4.5035	0.70711	15	0.0000
16	4.4936	0.00497	0.0068327	-5.3033	0.70711	16	0.0000
17	9.0532	0.0541	0.027726	-2.9167	0.70711	17	0.0000
18							

Figure S-1. Portion of KaleidaGraph data sheet for VF2 and VF3 analysis of LC-MS-MS data for cocaine and naltrexone from ref 11. The entries in the first two columns come from 5 replicates for each calibration x value.

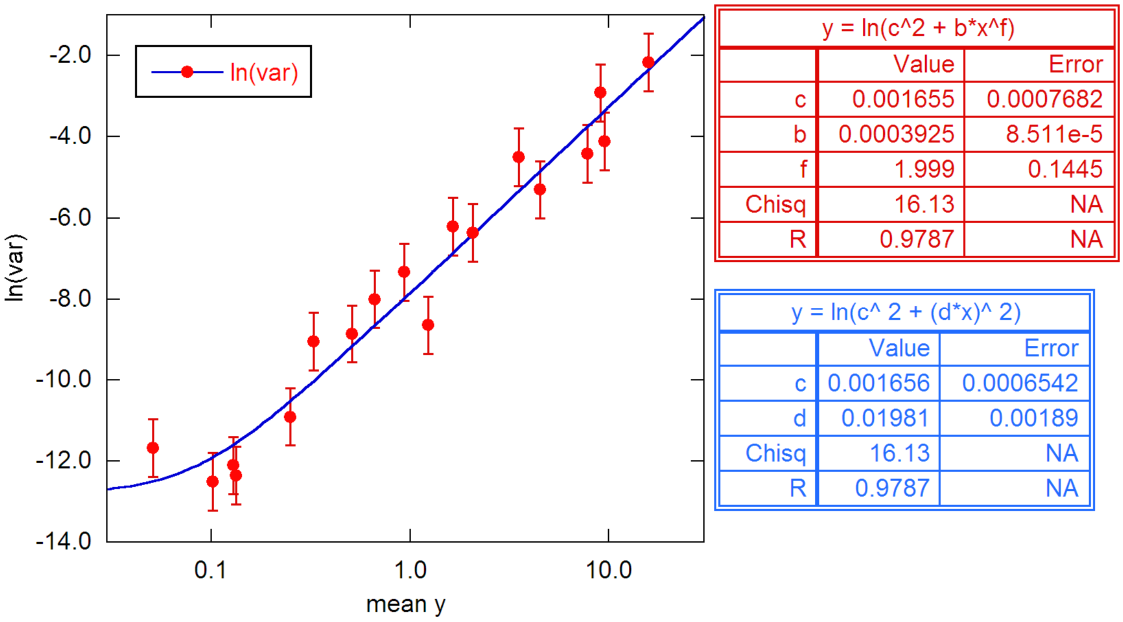

y = ln(c^2 + b*x^f)	Value	Error
c	0.001655	0.0007682
b	0.0003925	8.511e-5
f	1.999	0.1445
Chisq	16.13	NA
R	0.9787	NA

y = ln(c^ 2 + (d*x)^ 2)	Value	Error
c	0.001656	0.0006542
d	0.01981	0.00189
Chisq	16.13	NA
R	0.9787	NA

Figure S-2. Results for VF2 analysis with 3 adjustable parameters (red) and 2 (blue).

Results for VF3 analysis of these data are shown in Figure S-3. This method involves iterative reweighting, since the uncertainty is defined by the fitted VF, here σ = (c^2 + (d*y mean)^2)/sqrt(2). Initial σ values can be entered in **C2** using the command C2 = C1/sqrt(2) in the **Formula Entry** (**FE**) window (under the **Windows** menu). After the fit is run, the C2 entries are updated using the following command in the **FE** window:

C2 = (c^2 + (d*C0)^2)/sqrt(2). (S-3)

This procedure — fit and update C2 — is repeated until Chisq and the parameter values stabilize, which is typically ~10 cycles.

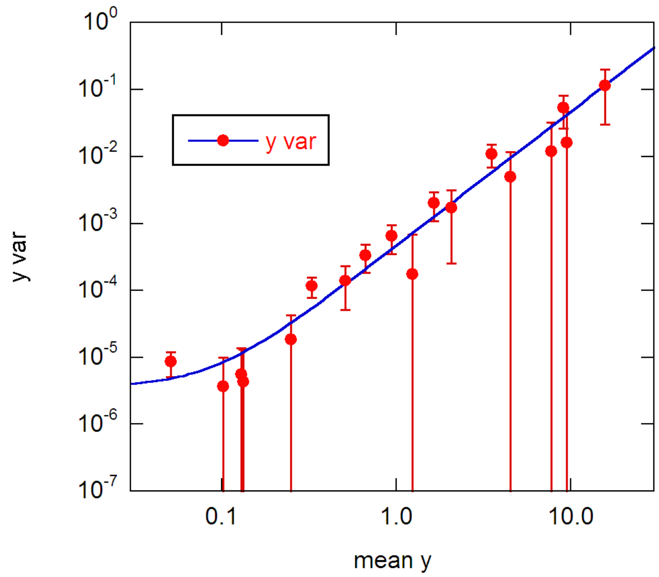

y = c^ 2 + (d*x)^ 2		
	Value	Error
c	0.00190212	0.000724766
d	0.0218693	0.00209212
Chisq	12.7376	NA
R	0.825765	NA

Figure S-3. VF3 analysis (iterative reweighting). Because the uncertainties are defined by the fit results, many of the s_i^2 values that are < VF have uncertainties exceeding their values, hence the long lower error bars.

To do the VF4 analysis with KG, we must use the **Macro Library** and display the points by their index number, with the plotted quantity being the residual/σ. Figure S-4 shows the library entries and the fit results for the equivalent of the 3-parameter fit in Figure S-2. Again the value of f is statistically consistent with the expected power 2, and replacing ff and bb as indicated yields c = 0.00231(67), d = 0.0253(21), and Chisq = 9.39, in agreement with results obtained using Excel below.

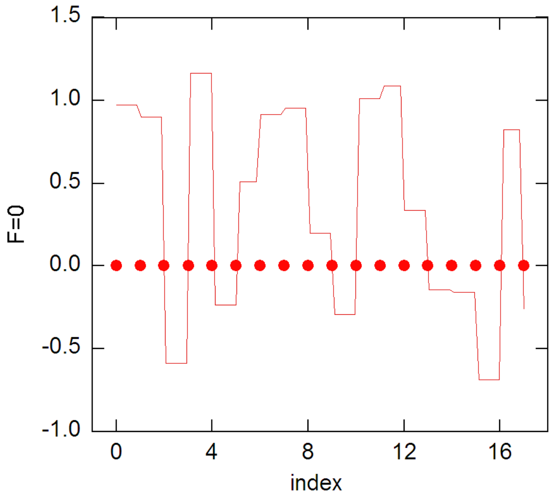


```
cc=c;
bb=b;
ff=f;
xx(x) = cell (x,0);
yy = cell(x,1);
var(x) = (cc^2 + bb*xx(x)^ff);
dof = 4;
sigt = (sqrt(2/dof)*var(x));
Fitfun = ((var(x)-yy)/sigt);
```

y = Fitfun		
	Value	Error
f	1.925	0.1367
c	0.002215	0.00079
b	0.0006588	0.000122
Chisq	9.217	NA
R	1	NA

Figure S-4. VF4 analysis. The use of statements like ff=f permits the parameters to be replaced with constants, *e.g.*, ff=2, or with other expressions, like bb=(d^2).

When a weighted fit is done with KG, the SEs are the *a priori* values,[8] which assume that the provided σ values are known absolutely. If they are known only relatively, the desired *post* SEs are obtained by scaling the provided SEs by (**Chisq**/ν)$^{1/2}$, where **Chisq** (chi-square,χ^2) is the sum of weighted squared residuals, provided in the fit output box. The χ^2 values in Figure S-2 are very close to ν = 16, so there is no need to convert the prior SEs to post. In S-3, the χ^2 value is not unreasonable; conversion to post involves scaling by 0.89. The algorithm used for S-4 yields the post SEs, and they (for f = 2, given above) agree with the Excel values for VF4 below. Note also that the *c* and *d* parameters increase by ~15% going from VF2 to VF3 and again from VF3 to VF5. This is consistent with the progressive lowering of χ^2. Also the *d*/*c* ratios are all within 5% of 11.5. This means that all three VFs would yield calibration parameters and their post SEs that are mutually statistically consistent.

The KG plot files for Figures S2-S4 are provided in a .zip file, along with those for Excel, discussed just below.

Excel Methods. The VF3 and VF4 methods can be implemented with just a slight modification of the worksheet, a portion of which is shown as Figure S-5 for VF3. Note that the sig(var) entries in column C are calculated using c' and d' in E10:E11. Solver is called to minimize the sum of squares (in B8) of the entries in F15:F32, by optimizing c and d in D3:D4. These must then be copied to E10:E11 for the next iteration. The values for SUMSQ in these iterations are shown in I; adequate convergence is obtained in 15 cycles. The VF4 implementation can be obtained by simply changing E10:E11 to =D3 and =D4. These results are shown in the indicated alternate worksheet in the Excel file.

The parameter SEs are obtained using the SolverAid routine from de Levie.[S1-S3] As used here, this routine gives post values, which for the VF4 method agree with results obtained in the KG 2-parameter version of Figure S-4 (see above). The SEs in Figure S-3 are prior; scaling them by the square root of the reduced chi-square, which appears in cell C8 in Figure S-5, brings them into agreement with the values in E3:E4.

The Excel file used here includes the SolverAid macro; to access it the user must click on "Enable Macros" when warned on opening it. Alternatively, the Macro can be downloaded from ref S-3.

	A	B	C	D	E	F	G	H	I	J	K	L
1												
2												
3			c =	0.00190087	*0.00064798*							
4			d =	0.02187114	*0.00186652*							
5												
6			CM:	*0.00000042*	*-0.00000035*							
7		**SolverAid**		*-0.00000035*	*0.00000348*							
8		12.7359	*0.892184*				**VF3 =**					
9							**EV solution**	sig(var) is defined by c' and d' in E10:E11.				
10				c' =	0.00190105			Solver optimizes on D3:D4, which must then be				
11				d' =	0.02187088			copied to E10:E11 for next iteration.				
12												
13	mean y	variance	sig(var)	calculated var	δ (calc-obs)	δ/σ	$(\delta/\sigma_{eff})^2$		Chi-square (B8), starting with *c*,*d* = 0.002,0.02.			
14												
15	0.12900	5.50E-06	8.18E-06	1.15735E-05	6.07E-06	7.42E-01	5.51E-01		16.8592			
16	0.24940	1.83E-05	2.36E-05	3.33666E-05	1.51E-05	6.39E-01	4.08E-01		12.9038			
17	0.32860	0.0001163	3.91E-05	5.52642E-05	-6.10E-05	-1.56E+00	2.44E+00		12.6926			
28	0.66300	0.0003345	0.000151	0.00021388	-1.21E-04	-7.98E-01	6.36E-01		12.7361			
29	0.93680	0.0006517	0.000299	0.000423408	-2.28E-04	-7.63E-01	5.81E-01		12.7359			
30	3.52700	0.01107	0.00421	0.005954117	-5.12E-03	-1.22E+00	1.48E+00					
31	4.49360	0.004975	0.006832	0.009662602	4.69E-03	6.86E-01	4.71E-01					
32	9.05320	0.05411	0.027724	0.039209119	-1.49E-02	-5.37E-01	2.89E-01					
33												
34	In C15: =(E10^2 + (E11*A15)^2)/sqrt(2) (copied to C16:C32)											
35	In D15: = D3^2 + (D4*A15)^2 (copied to D16:D32)					In B8: = SUMSQ(F15:F32)						
36	In E15: = D15-B15 (etc.)											
37	In F15: = E15/C15 (etc.)											

Figure S-5. Abbreviated Excel worksheet for VF3 analysis of data from ref 11.

References

S-1. de Levie, R. *Advanced Excel for Scientific Data Analysis*, 3rd ed., Atlantic Academic, LLC: Orrs Island, ME, 2012; available only from Amazon.com.

S-2. de Levie, R. Estimating Parameter Precision in Nonlinear Least Squares with Excel's Solver. *J. Chem. Educ.* **1999**, *76*, 1594-1598.

S-3. de Levie, R. Excellaneous: an ad-free, spyware-free web site for Excel users in the physical sciences. http://www.bowdoin.edu/~rdelevie/excellaneous.
